# Abdominal pain as first manifestation of lyme neuroborreliosis in children, case report and review of literature

**DOI:** 10.1186/s13052-020-00936-y

**Published:** 2020-11-23

**Authors:** Salvatore Savasta, Ivan Fiorito, Thomas Foiadelli, Anna Pichiecchio, Patrizia Cambieri, Bianca Mariani, Piero Marone, Gianluigi Marseglia

**Affiliations:** 1 Pediatric Clinic, Maggiore Hospital, ASST Crema, Crema, Italy; 2 Department of Pediatrics, Foundation IRCCS Policlinico San Matteo, University of Pavia, Pavia, Italy; 3 Neuroradiology Department, Foundation Casimiro Mondino, Pavia, Italy; 4 Virology Department, Foundation Policlinic San Matteo, Pavia, Italy

**Keywords:** Lyme neuroborreliosis, Abdominal pain, Radiculitis, Lyme disease, Radicular pain

## Abstract

**Background:**

Lyme neuroborreliosis can cause a variety of neurological manifestations. European children usually present facial nerve palsy, other cranial nerve palsies and aseptic meningitis.

**Case presentation:**

We hereby report a case of Lyme neuroborreliosis in a 9-year-old boy with abdominal pain as first symptom and subsequent onset of attention deficit and ataxia. Diagnosis was made by detection of specific antibody in both serum and cerebrospinal fluid with neuro-radiological images suggestive for this infectious disease. A 12-months follow-up was performed during which no relevant neurological sequelae were revealed.

**Conclusion:**

This case report shows that abdominal radiculitis, although extremely rare, could be the first manifestation of early Lyme neuroborreliosis in pediatric patients. Pediatricians must consider Lyme disease in the differential diagnosis of abdominal pain of unknown origin in children, especially in countries where the infection is endemic.

## Background

Lyme borreliosis (LB) is a clinically heterogeneous bacterial zoonosis caused by the spirochete *Borrelia burgdorferi* (Bb)*,* that is transmitted to humans by a tick bite [1]. LB is endemic in North America, Asia, central and northern Europe and is continually rising in Western Europe [1–3]. Incidence of infection peaks in 5–14 years old children and middle-aged adults (40–50 years old) [3]. Clinically, the illness develops in different sequential stages. The primary stage exhibits a peculiar skin rash (erythema migrans), a painless round shaped rash with slow enlargement that tends to resolve from the center that typically appears seven to 14 days after the bite. Fatigue or headache is often seen at this stage [1]. Blood or peripheral nerve dissemination of the bacterium lead to the subsequent stages, with various signs and symptoms. The secondary stage, that starts from three to 5 weeks after the bite, typically involves the neuronal or the cardiac tissue, usually causing atrioventricular blocks [1, 3]. The third and chronic stage is only rarely reached and is mainly represented by arthritis or late neurological complications [1, 4]. When the *Borrelia burgdorferi* sensu lato-complex invade the nervous system, the resulting clinical entity is called Lyme neuroborreliosis (LNB) [1, 3, 4]. Neurological symptoms usually occur between one and 12 weeks after the tick bite. Both the peripheral and central nervous systems can be affected by LNB. Cranial nerve neuritis, meningitis and radiculitis more often occur in the early period. Differently, late LNB usually presents with encephalitis, myelitis, encephalomyelitis and neuritis [1, 3, 5]. Early LNB, defined as signs and symptoms lasting less than 6 months after the tick bite, represent the vast majority of the cases (95%) [6]. While early LNB is typically self-limiting, late LNB is a chronic debilitating disease that probably reflects a persistent infection in nervous tissue [6]. Signs and symptoms of LNB can differ between distinct endemic areas, probably reflecting the geographical distribution of various genospecies of *Borrelia*, each of which has different neurotropic and neurovirulent properties [6]. The species involved in european Lyme disease are mostly *B. afzelii* and to a lesser extent *B. garinii* and *B. burgdorferi*. In the USA LNB is virtually due only to *B. burgdorferi* sensu stricto. Clinically, painful meningoradiculitis (Bannwarth’s syndrome) is the most common presentation of LNB in Europe [1, 3, 6, 7]. Patients with Bannwarth’s syndrome experience radicular pain, cranial nerve paresis and meningeal signs and symptoms [1, 3, 6, 7]. The pain is usually described as different from other previously experienced types of pain and is usually resistant to analgesic treatment [8, 9]. In the USA, erythema migrans and arthritis are more frequent compared to Europe and the main neurological manifestation is lymphocytic meningitis. Furthermore, LNB usually has different presentations according to age: in European children, the most common manifestation of LNB is acute facial nerve palsy, followed by other cranial nerve palsies and lymphocytic meningitis [6, 7]. Preschool children may present with unspecific symptoms such as loss of appetite and asthenia. Although rare, central nervous system involvement such as myelitis or encephalitis has been reported in children with LNB. In this case, it can manifest with a very wide (and non-specific) range of symptoms including anorexia, ataxia and attention deficit [6].

LNB poses a clinical diagnostic challenge which relies on the detection of blood-CSF-barrier dysfunction, pleocytosis and intrathecal production of immunoglobulins (Ig) and Bb-specific antibodies [6, 10]. The outcome is generally favorable if adequate antibiotic treatment is administered although in 15% of pediatric patients some long-term neurological deficits are found [11].

## Case presentation

A 9-year-old boy of Italian descent was referred to our Pediatric Department with a one-year history of abdominal pain, progressive poor scholastic performance and gait disturbance in absence of a clear diagnosis. At the age of 8 years and 2 months he had been hospitalized for severe abdominal pain of unknown origin. The stools were normal without history of constipation or diarrhea. All routine laboratory tests were normal. An abdominal ultrasonography, esophageal gastroduodenoscopy and colonoscopy with mucosal biopsies, and an abdominal CT-scan were sequentially performed, without evidences of any abnormality. Common causes of pediatric abdominal pain such as peritonitis, appendicitis, cholecystitis, pancreatitis, intra-abdominal abscesses, malignancy, inflammatory bowel disease, vasculitis and pleural abnormalities, malignancies, eosinophilic gastro-enterocolitis and inflammatory bowel disease were ruled out. Fecal cultures, parasitic examination and virus polymerase chain reaction test on stools all resulted negative. Family history was negative for psychiatric disorders, and absence of emotional or anxiety disorder was assessed by a pediatric psychiatrist.

After discharge the pain gradually and spontaneously remitted over the next 2 months. Six months before our evaluation he experienced learning difficulties with attention deficit and irritability, in addition, he developed difficulty in walking.

When he was admitted to our Department he presented with ataxic gait, difficulty in speaking and attention deficit. Physical examination was unremarkable except for bilateral patellar areflexia and sensory ataxia with positive Romberg sign. Routine laboratory values (including erythrocyte sedimentation rate, C-reactive protein level and white cell count) were normal. Nerve conduction studies of the lower limbs showed bilateral slowed conduction in the peroneal motor nerves. Brain and spine magnetic resonance imaging (MRI) showed a diffuse contrast enhancement of the leptomeninges and of the III, V and VII cranial nerves, as well as of the cauda equina nerve roots, with multiple bilateral T2-hyperintense lesions in periventricular white matter. CSF analysis showed lymphocytic pleocytosis (290 cells/mm3 – normal values < 5 cells/mm3), low glucose level (15 mg/dl - normal values > 50 mg/dl), hyperproteinorrachia (587 mg/dl - normal values < 45 mg/dl). Bacterial blood and CSF cultures were negative as well as a multiple molecular assay panel for meningitis and encephalitis (BIOFIRE® FILMARRAY® ME Panel, BioMerieux Italy) for the most common bacteria and viruses linked with encephalitis.

Angiotensin-converting enzyme (ACE) activity on serum and CSF was negative excluding neurosarcoidosis.

Antineuronal antibodies were negative (i.e. anti-NMDA-R, anti-AMPA-R, anti-GABA, anti-CASPR2, anti-Hu, anti-CV2, anti-RI, anti-Yo, anti-amphiphysin, anti-GAD, anti-Sox1). CSF oligoclonal IgG bands were found at immunofixation and Lyme neuroborreliosis was considered.

**Fig. 1:**
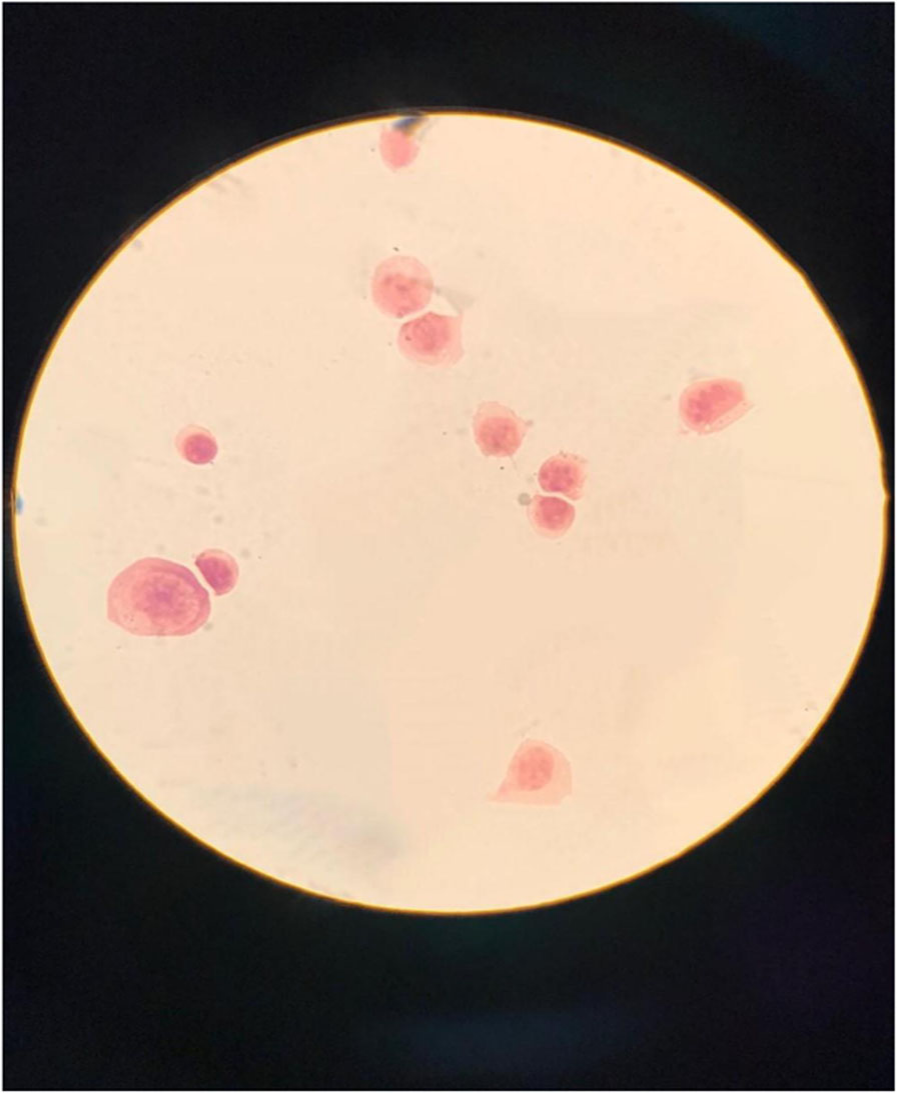
Cytospin of CSF. Activated mononuclear cells and polymorphonuclear cells

The Clinical Microbiology Laboratory used a cytospin slide centrifuge to prepare CSF Gram stains. Cytospin smears were prepared by centrifuging 0.2 mL of CSF at 2000 rpm for 10 min and staining it with conventional Gram staining, slides were examined by light microscopy at 100x magnification to allow quantitation of CSF cells: many activated mononuclear cells and some polymorphonuclear cells were identified, without evidence of bacteria or malignant cells (Fig. 1).

Serum *Borrelia burgdorferi*-specific IgG were detected (enzyme-linked immunosorbent assay ELISA followed by Western Blot positive for anti-VIsE and anti-p14, 21, 30). Specific antibodies in CSF were tested in an enzyme immunoassay (IDEIA Lyme Neuroborreliosis). *Borrelia burgdorferi* IgG index was 12.6 (references: < 0.3) and *B. burgdorferi* IgM index was 10.1 (references: < 0.3) which indicate intrathecal production of specific antibodies consistent with neuroborreliosis. Diagnosis of LNB was thus confirmed. Detection of *Borrelia burgdorferi*-DNA PCR resulted negative in the CSF. Indeed, a thorough past medical history collection evidenced that the onset of abdominal pain started 2 weeks after a tick-bite episode occurred during a walk in the wood. Neither the patient nor his parents showed any skin rash. According to the latest European clinical guidelines for LNB, the boy was treated with a course of intravenous ceftriaxone (3 g daily for 3 weeks) [6]. Furthermore the treatment was prolonged with oral amoxicillin (2 g daily for 3 weeks) considering the disseminated and long-lasting illness [12]. Three months thereafter, CSF findings had remarkably improved (20 cells/mm3, proteins 127 mg/dl, glucose 70 mg/dl) (Table 1). Similarly, we recorded a slight improvement in gait and better scholastic performances. At the one-year follow-up visit, complete neurological recovery was observed, without any recurrence of abdominal pain. The patient was considered completely healed, without specific signs and symptoms.

**Table 1:** Serologic and CSF results of the patient obtained on initial evaluation and at the follow-up visit

	At the diagnosis	At the diagnosis	3 months follow-up	3 months follow-up
	Serum	CSF	Serum	CSF
**Glucose**		15 mg/dl		50 mg/dl
**Proteins**		587 mg/dl		127 mg/dl
**Cells**		290 mm^3^		20 mm^3^
**Borrelia IgM ELISA**	Positive		Positive	
**Borrelia IgG Western Blot**	Positive		Positive	
**Borrelia PCR**		Negative		
**Borrelia** **Intrathecal antibody**		Positive		Positive

## Discussion and conclusion

Diagnosis of LNB is often difficult in areas with low disease prevalence such as in Italy where the estimated incidence is 1/100,000,000 [13]. In Europe, the most common strain associated to LNB is *Borrelia garinii*, and the disease usually presents with radicular pain and meningeal signs [5–7, 13]. Painful meningoradiculitis (Bannwarth’s syndrome) is more frequent in adults [5, 7, 13], while children usually display facial nerve palsy and headache due to meningitis [5–7]. We hereby present the challenging diagnosis of a unique pediatric case of LNB. In fact, at the first hospital admission and for several weeks, he presented abdominal pain as the only disease manifestation, without any additional sign of neurological involvement. At our observation and in light of a broader spectrum of central and peripheral neurological deficits that are typical for late LNB, we could speculate on the true radiculopathic nature of the initial symptom which was abdominal pain in our case. Although not confirmed by nerve conduction studies, the clinical characteristics of the pain, the exclusion of other causes, the diffuse spinal roots enhancement on MRI and the additional confirmation of peripheral neuropathy are highly suggestive for abdominal neuroradiculopathy as symptom of onset of the disease. We emphasize that our patient did not develop facial paralysis, which is typical in Bannwarth’s syndrome, instead we observed ataxic gait, learning difficulties with attention deficit and irritability, signs and symptoms reflecting bacterial involvement of central nervous system. These clinical characteristics, in addition to the serological and radiological findings, permitted us to establish the diagnosis of the late form of LNB [6].

To date we have no reports of cases from other authors with this symptoms of onset at pediatric age, therefore this is the first pediatric case with painful radiculitis causing isolated abdominal pain as the manifestation of onset of Lyme disease. Indeed, painful radiculopathy is rarely seen in children and abdominal neuropathic pain has never been described, even in association [5–9, 14], while it is occasionally described in adults [15–17]. Schwenkenbecher et al. in 2017 reported 17 cases of painful radiculitis mainly in back, arms and legs regions with the absence of abdominal in their retrospective study examining 68 patients with LNB of which 11 were children [18]. In a recent prospective multicentre study, only 6 out of 169 children with LNB had signs of meningoradiculitis with pain localized in back, in the arms or the legs, none of which presented with abdominal pain [7]. In another retrospective study on 143 children with LNB, only ten cases showed radicular pain, mainly localized in the head and neck region [5]. Even more strikingly, in the largest retrospective study published so far, no cases of meningoradiculitis were identified among 548 children with LNB [14].

Broekhuijsen-van Henten et al. in 2010, reported no cases of abdominal pain in children, only radicular pain mainly localized in neck, arm, leg, back and facial regions was found [19].

Finally, it is possible that radicular involvement may be underestimated in children, especially without complain of pain. In case of peripheral nerve involvement such as sensory ataxia and hypo−/areflexia, LNB should always be suspected. An early diagnosis of Lyme disease is crucial as targeted treatment can lead to fast clinical improvement with long term sequelae prevention [11].

## Data Availability

All data generated or analysed during this study are included in this published article.

## Abbreviations

LB: Lyme borreliosis
Bb: Borrelia burgdorferi
LNB: Lyme neuroborreliosis
ACE: Angiotensin-converting enzyme
CSF: Cerebral-spinal fluid
PCR: Polymerase chain reaction
CT: Computed tomography
NMDA-R: *N*-methyl-d-aspartate receptor
CASPR-2: Contactin-associated protein-like 2 receptor
AMPA-R: Anti-α-amino-3-hydroxy-5-methyl-4-isoxazolepropionic acid receptor
GABA: Gamma-aminobutyric acid
